# C-Reactive Protein and Long-Term Prognosis in Adult Patients with Congenital Heart Disease

**DOI:** 10.3390/jcm13082199

**Published:** 2024-04-11

**Authors:** Efrén Martínez-Quintana, María Alcántara-Castellano, Marta Isabel García-Suárez, Fayna Rodríguez-González

**Affiliations:** 1 Cardiology Service, Complejo Hospitalario Universitario Insular-Materno Infantil, Avd. Marítima del Sur s/n, 35016 Las Palmas de Gran Canaria, Spain; 2 Department of Medical and Surgical Sciences, Faculty of Health Sciences, Universidad de Las Palmas de Gran Canaria, 35016 Las Palmas de Gran Canaria, Spain; 3 Hospital Universitario de Gran Canaria Dr. Negrín, 35010 Las Palmas de Gran Canaria, Spain

**Keywords:** adult, congenital heart disease, survival, prognosis, cardiovascular outcome, C-reactive protein

## Abstract

**Background/Objectives**: Prognostic biomarkers may provide information about the patient’s cardiovascular outcomes. However, there are doubts regarding how high-sensitivity C-reactive protein (hs-CRP) impacts patients with congenital heart disease (CHD). The main objective is to evaluate whether high hs-CRP levels predict a worse prognosis in patients with CHD. **Methods**: Observational and prospective cohort study. Adult CHD patients and controls were matched for age and sex. **Results**: In total, 434 CHD patients (cases) and 820 controls were studied. The median age in the CHD patients was 30 (18–62) years and 256 (59%) were male. A total of 51%, 30%, and 19% of patients with CHD had mild, moderate, and great complexity defects, respectively. The body mass index [1.07 (1.01–1.13), *p* = 0.022)], diabetes mellitus [3.57 (1.07–11.97), *p* = 0.039], high NT-pro-BNP levels [1.00 (1.00–1.01), *p* = 0.021], and low serum iron concentrations [0.98 (0.97–0.99), *p* = 0.001] predicted high hs-CRP levels (≥0.3 mg/dL) in patients with CHD. During a follow-up time of 6.81 (1.17–10.46) years, major cardiovascular events (MACE) occurred in 40 CHD patients, showing the Kaplan–Meier test demonstrated a worse outcome among patients with hs-CRP levels above 0.3 mg/dL (*p* = 0.012). Also, hs-CRP showed statistical significance in the univariate Cox regression survival analysis. However, after adjusting for other variables, this significance was lost and the remaining predictors of MACE were age [HR 1.03 (1.01–1.06), *p* = 0.001], great complexity defects [HR 2.46 (1.07–5.69), *p* = 0.035], and an NT pro-BNP cutoff value for heart failure > 125 pg/mL [HR 7.73 (2.54–23.5), *p* < 0.001]. **Conclusions**: Hs-CRP obtained statistical significance in the univariate survival analysis. However, this significance was lost in the multivariate analysis in favor of age, CHD complexity, and heart failure.

## 1. Introduction

As more than 85% of the patients with congenital heart disease (CHD) survive into adulthood due to the advances in diagnostic testing, pediatric cardiac care, timely surgical interventions, and catheter-based interventions, nowadays, in industrial countries, there are more adults than children with CHD [1]. For this reason, it is becoming increasingly important to find diagnostic tools that allow us to determine the clinical outcomes of these patients.

C-reactive protein (CRP), a plasma protein synthesized by the liver, is a sensitive and dynamic systemic marker of inflammation [2]. As atherosclerosis is associated with inflammation within the vessel walls, CRP may also be an indicator of cardiovascular risk. Moreover, low-grade inflammation is a common feature in subjects with type 2 diabetes [3], and increasing evidence shows that CRP is not only an inflammatory biomarker but also an important risk factor associated with ageing-related diseases [4].

Despite the fact that CRP has been related to coronary heart disease, ischemic stroke, and cardiovascular mortality, clinical investigations suggest that ischemic vascular events depend considerably on conventional risk factors and other markers of inflammation [5]. In fact, there are doubts about the direct association between CRP levels and cardiovascular outcomes as some authors conclude that this association is more likely to be explained by confounding factors seen in observational studies or the effects of treatments in clinical trials [6].

Elevated CRP has also been associated with adverse clinical outcomes in adults with CHD and some authors advise routine clinical assessment, in addition to NT-pro-brain natriuretic peptide (NT-pro-BNP) measurement, to improve risk stratification [7]. Also, other authors have reported that adults with CHD and elevated CRP not only have a greater risk for death or non-elective cardiovascular hospitalization but also a worse functional status and exercise capacity [8]. Furthermore, serial CRP measurements seem to provide prognosis after hospital discharge, identifying CHD patients at higher risk of re-admission for heart failure [9].

The aim of this study is (a) to compare CRP levels in CHD patients and a control group; (b) to analyze which variables influence higher CRP concentrations among patients with CHD, and (c) to evaluate whether high levels of CRP lead to a worse cardiovascular outcome in patients with CHD.

## 2. Methods

This was an observational, analytic, prospective cohort study design. The case group was made up of consecutive CHD patients, over 18 years old, seen in a single adult CHD outpatient unit between January 2007 and December 2018. The controls were obtained from patients older than 18 years attending community health centers in the same geographical area between July 2017 and December 2018 for health promotion or disease prevention. The controls were matched for age and sex to patients with CHD. Those patients who did not give written informed consent to participate or who had surgery or hospital admission in the previous six months were excluded from the research. Ethics approval was obtained from our hospital Research Ethics Committee.

### 2.1. Clinical Data

The existence of CHD was determined by imaging studies, preferably echocardiography, and the anatomic complexity was classified as simple, moderate, or great cardiac defects [10]. The cardiovascular risk factors, which included arterial hypertension, diabetes mellitus, dyslipidemia, and a smoking habit, were defined as previously reported [11]. The body mass index (BMI) was derived from the person’s weight in kilograms (kg) and the height in meters (m) using the formula BMI = kg/m^2^. The glomerular filtration rate was estimated using the Modification of Diet in Renal Disease Study equation [12]. Medical treatment included anticoagulant and antiplatelet therapy, antihypertensive medication, and iron supplements. Among the patients with CHD, having atrial fibrillation or a flutter was determined by electrocardiogram. Meanwhile, suffering from rheumatic or autoimmune diseases or being a carrier of a mechanical valve prosthesis was obtained by reviewing the medical records. Systemic ventricular dysfunction was defined, by echocardiogram, as a left ventricular ejection fraction < 40% or a tricuspid annular plane systolic excursion (TAPSE) < 17 mm if the right ventricle supported the systemic circulation [13]. A level of 125 pg/mL was used as the cutoff point for NT-pro-BNP because it effectively rules out left ventricular dysfunction [14]. Meanwhile, a high-sensitivity C-reactive protein (hs-CRP) level of less than 0.3 mg/dL was considered normal as it is seen in most healthy adults [15].

### 2.2. Blood Test

Blood samples were obtained for research purposes, with informed consent from the subjects, after an overnight fast of at least 10 h. The tested analytes were obtained by spectrophotometry using an Olympus AU 2700 (Olympus Diagnostic, Hamburg, Germany). The hs-CRP levels were determined by the immunoturbidimetric method applied via the Olympus AU 2700 biochemistry analyzer. The regents and calibrators were used according to the manufacturers’ recommendations with analytic measuring ranges of 0.05–20 mg/dL for the hs-CRP test. Finally, the NT-pro-BNP levels were measured by immunoassay using the Siemens Stratus CS Acute Care Diagnostic System (Siemens Healthcare Diagnostics, Inc., Newark, DE, USA). Biochemistry analyzers and the same reference values were used for all the CHD and control patients.

### 2.3. Follow-Up

A MACE (major adverse cardiac event) was defined as a composite of nonfatal stroke, nonfatal myocardial infarction, and cardiovascular mortality [16]. The follow-up time was measured from the start of the study until the first MACE occurred. The clinical history and the International Classification of Diseases (ICD) data from our hospital were used to categorize diseases and determine MACE occurrence.

### 2.4. Statistical Analysis

The parametric data are presented as the mean and standard deviation (±) and the non-parametric data as the median and 5–95 percentiles. The Kolmogorov–Smirnov test was used to test the null hypothesis that a set of data comes from a normal distribution. Pearson’s χ^2^ test was used to assess the difference in the distribution of a categorical variable between two or more independent groups, and the non-parametric data were compared with the use of the Mann–Whitney rank-sum test.

Logistic regression analysis was used to examine the association of (categorical or continuous) independent variables with one dichotomous dependent variable. For this purpose, the hs-CRP concentration was classified in a binary manner (above or below 0.3 mg/dL). The covariates that showed significance in the univariate analysis (*p* < 0.05) were entered into the regression analysis after observing the association between them to determine which ones were finally included in the regression analysis. By default, the method that was selected for performing the regression analysis was the enter method. Effect estimates were reported along with the odds ratio (OR) value, 95 % confidence intervals (CI), and *p* value. The crude OR was obtained after considering the effect of only one predictor variable, and the adjusted OR was determined after including all the variables that showed significance in the crude odds ratio analysis. Odds ratios greater than 1 correspond to “positive effects” because they increase the odds. Those between 0 and 1 correspond to “negative effects” because they decrease the odds. Odds ratios of exactly 1 correspond to “no association”.

The Kaplan–Meier curve was used to analyze the time from inclusion to any MACE depending on whether the hs-CRP levels were above or below 0.3 mg/dL and the log-rank tested the differences. Cox regression analysis was used to assess the association between the variables and the survival rate. Cox regression generated hazard ratios (HRs), which were interpreted with a 95% CI. In a Cox model, the HR represents the relative risk of the event occurring for a given unit change in the predictor variable, with an HR greater than 1 indicating an increased risk and an HR less than 1 indicating a decreased risk. The Statistical Package for the Social Sciences (SPSS 24, Chicago, IL, USA) was used for data analysis.

## 3. Results

### 3.1. Study Population

In total, 434 patients with CHD who were followed up in our outpatient unit signed the informed consent and were included in the study. Meanwhile, the control group was formed by 820 patients. According to the anatomic complexity, patients were classified as having simple [222 (51%) patients], moderate [131 (30%) patients], and great [81 (19%) patients] defects, as shown in Table 1. Sixteen (4%) patients with CHD were carriers of a mechanical valve prosthesis, fifteen (3%) had systemic ventricular dysfunction, and twenty (5%) patients showed atrial fibrillation or a flutter in the electrocardiogram. On the contrary, none of them suffered from rheumatic or autoimmune diseases.

#### 3.1.1. Clinical and Blood Test Data in Patients with CHD and the Control Population

From a clinical point of view, smokers were significantly more frequent in the control group than in the patients with CHD. Nonetheless, no significant differences were found between both groups in arterial hypertension, diabetes mellitus, or dyslipidemia. In relation to the medication, patients with CHD were more frequently under antiplatelet therapy, oral anticoagulants, beta-blockers, angiotensin-converting enzyme (ACE) inhibitors and angiotensin receptor blockers (ARBs), calcium channel blockers, and loop diuretics than patients in the control group (*p* < 0.05). On the contrary, no statistical significance was seen in oral iron or statin treatment between both groups. Regarding the blood test, as can be seen in Table 2, the patients with CHD had higher creatinine and bilirubin concentrations than the patients in the control group. In total, 287 out of 820 control patients (35%) and 153 out of 434 (35.2%) patients with CHD had hs-CRP concentrations above 0.3 mg/dL (*p* = 0.679).

#### 3.1.2. Clinical and Blood Test Data in CHD Patients according to Their Hs-CRP Levels

The patients with CHD and a hs-CRP concentration ≥ 0.3 mg/dL were significantly older, had a higher BMI, and were more diabetic and dyslipidemic than the patients with a hs-CRP below 0.3 mg/dL (*p* < 0.05). Similarly, the patients with higher hs-CRP levels had a worse NYHA functional class and showed more frequent atrial fibrillation or flutter in the electrocardiogram than the CHD patients with lower hs-CRP levels (<0.3 mg/dL). In relation to the blood test, the CHD patients with high hs-CRP levels (≥0.3 mg/dL) had significantly lower hemoglobin, bilirubin, and iron levels than the patients with hs-CRP concentrations below 0.3 mg/dL. On the other hand, as seen in Table 3, those CHD patients with higher hs-CRP concentrations showed higher alanine aminotransferase (ALT) and NT pro-BNP levels than the patients with lower hs-CRP concentrations. In relation to the occurrence of MACE, the CHD patients with higher hs-CRP concentrations (≥0.3 mg/dL) had a significantly higher incidence of myocardial infarctions and cardiovascular mortality than the CHD patients with lower hs-CRP levels (*p* < 0.05). On the contrary, no significant differences were seen according to stroke, regardless of the hs-CRP concentrations (Table 3).

### 3.2. Predictors of High CRP Levels in Patients with CHD

Table 4 shows the variables that predicted high hs-CRP concentrations in patients with CHD. The results of the logistic regression analysis are presented as both the unadjusted (or crude) odds ratios based on a simple model with only one variable at a time and the adjusted odds ratios for a model with all the variables that showed significance. As can be seen in the table in the odds ratio adjusted column, those variables that predicted high hs-CRP levels (≥0.3 mg/dL) in the CHD group were the BMI [1.07 (1.01–1.13), *p* = 0.022)], being diabetic [3.57 (1.07–11.97), *p* = 0.039], and having higher NT-pro-BNP levels [1.00 (1.00–1.01), *p* = 0.021] and lower serum iron concentrations [0.98 (0.97–0.99), *p* = 0.001].

### 3.3. MACE in Patients with CHD

**Figure 1:**
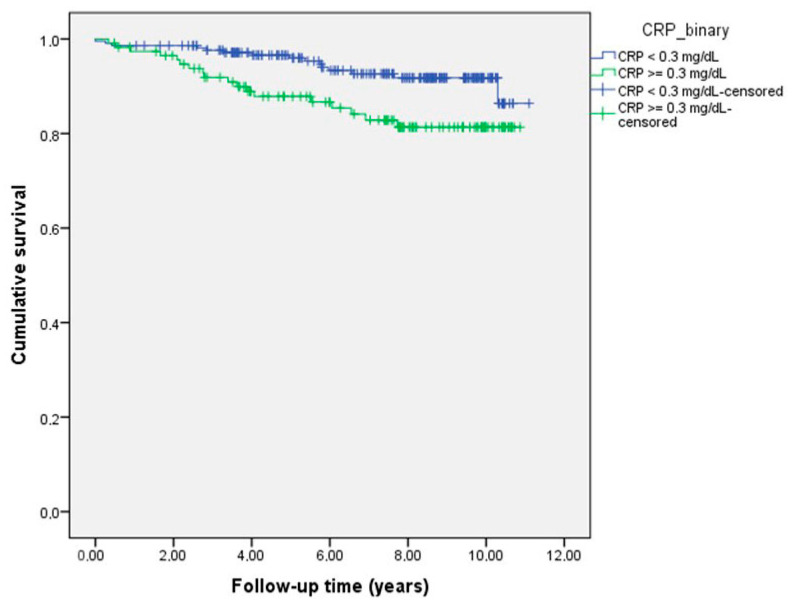
Kaplan–Meier curve showing major adverse cardiovascular events in congenital heart disease (CHD) patients with CRP levels above (blue line) and below (green line) 0.3 mg/dL (*p* = 0.012 for the log-rank test).

The patients with CHD were followed up during a median time of 6.81 (1.17–10.46) years. MACE occurred in 40 patients with CHD. In total, 5 patients had myocardial infarction, 15 had stroke, and 20 patients died due to cardiovascular events. Significant differences were found between myocardial infarction (*p* = 0.040), cardiovascular mortality (*p* = 0.006), and MACE (*p* = 0.009) in CHD patients with hs-CRP levels above and below 0.3 mg/dL (Table 3). As can be seen in Figure 1, the Kaplan–Meier test showed that patients with CHD and a hs-CRP concentration above 0.3 mg/dL had a significantly worse outcome than CHD patients with hs-CRP levels below 0.3 mg/dL (*p* = 0.012 for the log-rank test).

Meanwhile, the multivariate Cox regression analysis (Table 5) carried out in patients with CHD showed an independent association between age [HR 1.03 (1.01–1.06), *p* = 0.001], great CHD complexity [HR 2.46 (1.07–5.69), *p* = 0.035] and NT-pro-BNP concentration (>125 pg/mL) [HR 7.73 (2.54–23.5), *p* < 0.001], and the occurrence of MACE. However, the hs-CRP concentration lost its univariate significance after adjusting for the rest of the variables included in the survival analysis.

## 4. Discussion

CRP is one of multiple markers of inflammation seen in atherosclerosis and other cardiovascular diseases, which involves low-grade systemic inflammation. Clinical interest in these markers has focused on their potential utility in predicting future cardiovascular events and, thereby, in patient management.

Hs-CRP levels less than 0.3 mg/dL are considered normal as these levels are seen in most healthy adults [15]. However, in our study, 35% of our patients, both in the control and the case groups, showed hs-CRP levels above 0.3 mg/dL, which is higher than the percentages found in other control (31.1% in non-Hispanic whites among the USA adult population) [17] and CHD (25 to 28% in adult CHD patients) groups [7,8]. These findings may be explained by the higher percentage of diabetes mellitus seen in our series (5.7% vs. 3.5%) when compared with the results found by Opotowsky et al. [8], despite the younger age (29 ± 14 vs. 38 ± 15 years old) and the lower BMI (24 ± 6 vs. 27 ± 6 kg/m^2^) of our CHD patients. Type two diabetes mellitus is characterized by a chronic inflammation status, and the production of CRP may be triggered by many metabolic and inflammatory factors associated with the development of diabetes mellitus, such as increased blood glucose, adipokines, and free fatty acid levels [18]. In fact, low-grade systemic inflammation has been associated with an increased risk of diabetes mellitus in middle-aged patients [19] and is an independent predictor [2,20].

In relation to the clinical and blood test variables, which were revealed to be predictors of high hs-CRP concentrations, we found that diabetes mellitus, BMI, NT-pro-BNP concentration, and iron levels reached statistical significance. On the one hand, subclinical inflammation, which is found in type 2 diabetic patients, is characterized by elevated circulating levels of inflammatory markers and obesity and involves an excess of macronutrients in the adipose tissue, which favors the release of inflammation mediators such as interleukin 6 which stimulates the liver to synthesize and secrete CRP [21]. In fact, weight loss has been strongly associated with reductions in circulating hs-CRPs [22]. In relation to NT-pro-BNP levels, a valuable predictor in the diagnosis and prognosis of patients with symptoms of heart failure, it has long been recognized that heart failure may manifest some of the clinical features observed in chronic inflammatory conditions. Moreover, it is thought that several proinflammatory cytokines may be involved in the pathogenesis of ventricular dysfunction [23]. On the other hand, iron deficiency is very common but often overlooked in people with chronic conditions [24] such as autoimmune diseases, cancer, chronic infections, or inflammatory bowel disease [25], and iron, and its homeostasis, is intimately tied to the inflammatory response [26].

Regarding clinical outcome and hs-CRP levels, we obtained a significant association between having high hs-CRP concentrations and myocardial infarction and cardiovascular mortality, which is in line with events seen in other populations at low or intermediate risk of cardiovascular events. Nonetheless, the evidence base supporting the inclusion of CRP in vascular disease risk assessment is incomplete and sometimes conflicting [27]. Some meta-analyses, carried out in the general population, suggest that the risk of cardiovascular events rises with increasing CRP levels [28]. On the contrary, other authors have concluded that the relevance of CRP with the risk of ischemic vascular disease is unclear and depends considerably on conventional risk factors and other markers of inflammation [5].

Among patients with CHD, Geenen et al. [7] found in a prospective cohort study of 602 adult patients with CHD that hs-CRP carried out an incremental prognostic value for the risk of death or heart failure, beyond the NT-pro-BNP concentration. Moreover, they observed that hs-CRP increased prior to the occurrence of heart failure or death, supporting the role of inflammation in the clinical deterioration of patients with CHD. On the contrary, Opotowsky et al. [8] did not obtain statistical significance between the hs-CRP concentration and a history of coronary artery disease or cerebrovascular accident. As the authors state, this could first be explained by the low prevalence of these diseases and second, by the fact that many of the patients were under antiplatelet and anticoagulation pharmacotherapy. In our series, we found an association between hs-CRP and cardiovascular events in adult patients with CHD, which is in line with events seen in other low-risk cardiovascular populations [29]. However, when the Cox regression analysis included multiple predictors, the significance of the hs-CRP concentrations was lost in favor of other powerful factors, such as age, the CHD complexity, or the NT-pro-BNP levels, as also seen in the general population [30] and in patients with CHD [7].

### Limitations

However, there may be limitations to the study that may have affected our results. First, the occurrence of concomitant infections or inflammatory processes may have led to increasing hs-CRP levels. However, none of our patients with CHD suffered from rheumatic or autoimmune diseases and only those seen in routine medical check-ups were included in the analysis, therefore, making the interference of infections or inflammatory processes with CRP measurement unlikely. Likewise, we excluded hospitalized or operated-on CHD patients, which further reduces this possibility. Second, we did not record the use of oral contraceptives or hormonal replacement among our female patients as these drugs tend to elevate CRP levels. However, we found no significant differences in the hs-CRP concentrations between male and female patients in the CHD group. Also, the low number of MACE observed during the follow-up time, typical of young populations, reduced the possibility of obtaining a greater number of events. Nonetheless, we believe that our sample is large enough to establish a link between hs-CRP concentrations and cardiovascular outcomes in patients with CHD. Finally, patients with CHD represent a heterogeneous population so it may be difficult to provide firm conclusions about their outcomes. Despite these limitations, we believe that the number of patients with CHD included in our series and the possibility of comparison with a control population allows us to shed light on the usefulness of CRP concentration as a predictor of cardiovascular events in patients with CHD.

In conclusion, we found in our series, both in the CHD and control groups, a high prevalence of patients with high hs-CRP concentrations (35%) that is higher than the prevalence seen in previous studies. Also, we determined that having high hs-CRP levels was a predictor of diabetes mellitus, as also occurs in the normal population. Finally, although the hs-CRP concentration was a predictor of survival in the univariate Cox regression analysis, after including well-known clinical factors such as age, CHD complexity, or the NT pro-BNP levels, this significance was lost.

## Figures and Tables

**Table 1:** Congenital heart disease classification according to complexity.

Types of CHD according to Complexity	Number of Patients
**Simple complexity**	222
Aortic valve disease	26
Pulmonary valve disease	41
Atrial septal defect	49
Ventricular septal defect	70
Ductus	10
Anomalous pulmonary venous drainage	2
Other simple defects	24
**Moderate complexity**	131
Subvalvular or supravalvular aortic stenosis	15
Coarctation of the aorta	39
Subvalvular or supravalvular pulmonary stenosis	7
Tetralogy of Fallot	37
Ebstein	5
Atrioventricular septal defects	28
**Great complexity**	81
Dextro transposition of the great arteries	17
Levo transposition of the great arteries	9
Pulmonary atresia	4
Single ventricle	10
Double outlet right ventricle	7
Tricuspid atresia	3
Trucus arteriosus	2
CHD with pulmonary arterial hypertension (Eisenmenger)	29
**Total of patients with CHD**	434

CHD: congenital heart disease.

**Table 2:** Demographic, clinical, and analytical data in patients with CHD and the control population.

	Control	CHD	*p* *
CHD patients, n	820	434	
Age, years	33 (19–49)	30 (18–62)	0.702
Sex (male), n	515 (63)	256 (59)	0.186
Arterial hypertension, n	83 (10)	59 (14)	0.065
Diabetes mellitus			0.232
Type 1, n	8 (1)	3 (1)	
Type 2 diabetes oral hypoglycemic agents, n	29 (4)	15 (3)	
Type 2 with insulin, n	4 (0.5)	7 (2)	
Dyslipidemia, n	182 (22)	81 (19)	0.144
Smoking, n	152 (19)	23 (5)	<0.001
Laboratory results			
Glucose, mg/dL	94 (81–120)	94 (81–117)	0.134
Creatinine, mg/dL	0.8 (0.5–1.0)	0.9 (0.6–1.2)	<0.001
GFR, mL/min/1.73 m^2^	111 (83–153)	91 (61–154)	<0.001
Hemoglobin, mg/dL	14 (12–17)	15 (12–17)	0.083
Total bilirubin, mg/dL	0.6 (0.3–1.5)	0.7 (0.3–2.1)	<0.001
Total cholesterol, mg/dL	176 (122–246)	160 (108–231)	0.678
LDL cholesterol, mg/dL	101 (60–156)	91 (46–149)	0.634
HDL cholesterol, mg/dL	50 (35–75)	48 (32–70)	0.341
ALT, IU/L	19 (10–64)	17 (9–50)	0.847
AST, IU/L	22 (15–45)	22 (14–42)	0.702
Hs-CRP, mg/dL	0.17 (0.03–1.39)	0.16 (0.00–1.59)	0.404
Medical treatment			
Antiplatelet, n	9 (1)	40 (9)	<0.001
Oral anticoagulation, n	4 (0.5)	65 (15)	<0.001
Betablockers, n	18 (2)	62 (14)	<0.001
ACE inhibitors/ARBs, n	66 (8)	65 (15)	<0.001
Calcium channel blockers, n	11 (1)	16 (4)	0.006
Loop diuretics, n	25 (3)	61 (14)	<0.001
Oral iron, n	31 (4)	19 (4)	0.607
Statins, n	51 (6)	37 (9)	0.128

CHD: congenital heart disease, n: number of patients, GFR: glomerular filtration rate, ALT: alanine aminotransferase, AST: aspartate aminotransferase, s-CRP: high-sensitivity C-reactive protein, ACE: angiotensin-converting enzyme, ARBs: angiotensin receptor blockers. The data are expressed as medians and (5–95) percentiles and as numbers and percentages. * Categorical variables are evaluated using the Pearson chi-square test, continuous data with a normal distribution are compared using the Student´s *t*-test, and continuous data without a normal distribution are compared using the Mann–Whitney test.

**Table 3:** Demographic, clinical, and blood test data in CHD patients according to hs-CRP levels.

	CHD Patients		*p* *
	Hs-CRP < 0.3 mg/dL	Hs-CRP ≥ 0.3 mg/dL	
CHD patients, n	277	157	
Age, years	28 (19–61)	37 (19–64)	<0.001
Sex (male), n	172 (62)	84 (54)	0.080
BMI, kg/m^2^	23 (18–33)	25 (17–38)	<0.001
Great CHD complexity, n			0.131
Mild	147 (53)	73 (46)	
Moderate	86 (31)	47 (30)	
Great	44 (16)	37 (24)	
NYHA functional class (≥2), n	36 (13)	42 (27)	0.005
Arterial hypertension, n	33 (12)	26 (17)	0.175
Diabetes mellitus, n	7 (3)	18 (11)	<0.001
Dyslipidemia, n	42 (15)	39 (25)	0.013
Smoker, n	16 (6)	7 (4)	0.379
Laboratory results			
Glucose, mg/dL	93 (82–111)	94 (77–139)	0.267
Creatinine, mg/dL	0.9 (0.6–1.2)	0.9 (0.6–1.3)	0.247
GFR, mL/min/1.73 m^2^	91 (67–152)	90 (54–162)	0.289
Hemoglobin, mg/dL	15 (12–17)	14 (11–18)	0.017
Total bilirubin, mg/dL	0.7 (0.3–2.0)	0.6 (0.3–2.5)	0.016
Total cholesterol, mg/dL	156 (108–231)	166 (108–231)	0.130
LDL cholesterol, mg/dL	90 (46–148)	93 (47–152)	0.078
HDL cholesterol, mg/dL	50 (34–70)	47 (29–69)	0.070
ALT, IU/L	16 (9–48)	19 (10–54)	0.019
AST, IU/L	22 (14–39)	22 (14–43)	0.207
NT-pro-BNP, pg/mL	58 (0–712)	106 (6–1796)	<0.001
Iron, µg/dL	85 (29–157)	67 (18–133)	<0.001
Ferritin, ng/mL	34 (6–184)	38 (7–245)	0.250
Treatment			
Antiplatelet, n	22 (8)	18 (11)	0.223
Oral anticoagulation, n	31 (11)	34 (22)	0.035
Beta-blockers, n	30 (11)	32 (20)	0.006
ACE inhibitors/ARBs, n	33 (12)	32 (20)	0.018
Calcium channel blockers, n	9 (3)	7 (4)	0.521
Loop diuretics, n	26 (9)	35 (22)	<0.001
Statins, n	21 (8)	10 (6)	0.350
Oral iron, n	7 (2)	12 (8)	0.012
Mechanical valve prosthesis, n	11 (4)	5 (3)	0.670
Systemic ventricular dysfunction ^#^, n	6 (2)	9 (6)	0.057
Atrial fibrillation/flutter, n	7 (2)	13 (8)	0.006
Stroke, n	10 (4)	5 (3)	0.816
Myocardial infarction, n	1 (0.4)	4 (3)	0.040
Cardiovascular mortality, n	7 (3)	13 (8)	0.006
MACE, n	18 (6)	22 (14)	0.009

CHD: congenital heart disease, Hs-CRP: high-sensitivity C-reactive protein, n: number of patients, BMI: body mass index, NYHA: New York Heart Association, GFR: glomerular filtration rate, ALT: alanine aminotransferase, AST: aspartate aminotransferase, NT-pro-BNP: NT-pro-brain natriuretic peptide, ACE: angiotensin-converting enzyme, ARBs: angiotensin receptor blockers, ^#^ moderate to severe systemic ventricular dysfunction, MACE: major adverse cardiovascular events. The data are expressed as the medians and (5–95) percentiles and as numbers and percentages. * Categorical variables are evaluated using the Pearson chi-square test, continuous data with a normal distribution are compared using the Student´s *t*-test, and continuous data without a normal distribution are compared using the Mann–Whitney test.

**Table 4:** Binary logistic regression analyses in CHD patients to predict high hs-CRP levels.

	OR (Crude) (95% CI)	*p*	OR (Adjusted) (95%CI)	*p*
Age, years	1.09 (1.04–1.14)	<0.001	1.01 (0.99–1.04)	0.354
BMI, kg/m^2^	1.09 (1.04–1.14)	<0.001	1.07 (1.01–1.13)	0.022
NYHA (≥2)	2.24 (0.91–5.53)	0.080		
Diabetes mellitus	4.99 (2.04–12.24)	<0.001	3.57 (1.07–11.97)	0.039
Dyslipidemia	1.85 (1.13–3.01)	0.014	0.62 (0.23–1.70)	0.346
Hemoglobin, mg/dL	0.93 (0.84–1.03)	0.193		
Bilirubin, mg/dL	1.07 (0.94–1.21)	0.289		
NT-pro-BNP, pg/mL	1.01 (1.00–1.01)	0.005	1.00 (1.00–1.01)	0.021
Iron, µg/dL	0.99 (0.98–0.99)	0.001	0.98 (0.97–0.99)	0.001
ALT, IU/L	1.07 (0.98–1.02)	0.164		
Atrial/flutter fibrillation	3.48 (1.36–8.92)	0.009	1.02 (0.21–5.01)	0.980

CHD: congenital heart disease, Hs-CRP: high-sensitivity C-reactive protein, BMI: Body Mass Index, NYHA: New York Heart Association, NT-pro-BNP: NT-pro-brain natriuretic peptide, ALT: alanine aminotransferase, OR: odds ratio, CI: confidence interval.

**Table 5:** Univariate and multivariate Cox regression analysis of variables associated with major adverse cardiovascular events in patients with CHD.

	Univariate Analysis		Multivariate Analysis	
	HR (95% CI)	*p*	HR (95%CI)	*p*
Age, years	1.05 (1.03–1.07)	<0.001	1.04 (1.014–1.06)	0.001
CHD complexity ^a^	6.17 (3.04–12.51)	<0.001	2.46 (1.07–5.69)	0.035
NYHA class ^b^	7.13 (3.19–15.39)	<0.031	2.07 (0.81–5.22)	0.125
Diabetes mellitus	2.45 (0.91–6.60)	0.076		
NT pro-BNP ^c^	15.87 (5.57–45.22)	<0.001	7.73 (2.54–23.50)	<0.001
Hs-CRP ^d^	2.55 (1.28–5.09)	0.005	1.34 (0.56–3.19)	0.512

CHD: congenital heart disease, NYHA: New York Heart Association functional class, NT-pro-BNP: NT-pro-brain natriuretic peptide, Hs-CRP: high-sensitivity C-reactive protein. HR: Hazard ratio, CI: confidence interval. CHD complexity, NYHA, NT pro-BNP, and CRP are treated as binary variables: ^a^ CHD mild and moderate vs. great complexity, ^b^ NYHA class I vs. patients with class ≥ 2, ^c^ NT pro-BNP if levels were below 125 or ≥125 pg/mL, and ^d^ CRP if concentrations were < or ≥3 mg/dL.

## Data Availability

The participants of this study did not give written consent for their data to be shared publicly, so due to the sensitive nature of the research supporting data is not available.
